# Severe community-acquired *Enterobacter *pneumonia: a plea for greater awareness of the concept of health-care-associated pneumonia

**DOI:** 10.1186/1471-2334-11-120

**Published:** 2011-05-11

**Authors:** Alexandre Boyer, Brice Amadeo, Frédéric Vargas, Ma Yu, Sylvie Maurice-Tison, Véronique Dubois, Cécile Bébéar, Anne Marie Rogues, Didier Gruson

**Affiliations:** 1 Medical Intensive Care Unit, Hôpital Pellegrin-Tripode, Place Amélie Raba Léon, 33076 Bordeaux cedex, France; 2 Université de Bordeaux, INSERM U657, F-33000 Bordeaux, France; 3 INSERM, U657, F-33000 Bordeaux, France; 4 Institut de Santé Publique d'Epidémiologie et de Développement, Université de Bordeaux 2, 146, rue Léo Saignat, 33076 Bordeaux cedex, France; 5 Service de Bactériologie, Hôpital Pellegrin-Tripode, Place Amélie Raba Léon, 33076 Bordeaux cedex, France

**Keywords:** health-care-associated pneumonia, community-acquired pneumonia, *Enterobacter cloacae*, *Enterobacter aerogenes*, Gram-negative pneumonia

## Abstract

**Background:**

Patients with *Enterobacter *community-acquired pneumonia (EnCAP) were admitted to our intensive care unit (ICU). Our primary aim was to describe them as few data are available on EnCAP. A comparison with CAP due to common and typical bacteria was performed.

**Methods:**

Baseline clinical, biological and radiographic characteristics, criteria for health-care-associated pneumonia (HCAP) were compared between each case of EnCAP and thirty age-matched typical CAP cases. A univariate and multivariate logistic regression analysis was performed to determine factors independently associated with ENCAP. Their outcome was also compared.

**Results:**

In comparison with CAP due to common bacteria, a lower leukocytosis and constant HCAP criteria were associated with EnCAP. Empiric antibiotic therapy was less effective in EnCAP (20%) than in typical CAP (97%) (p < 0.01). A delay in the initiation of appropriate antibiotic therapy (3.3 ± 1.6 vs. 1.2 ± 0.6 days; p < 0.01) and an increase in duration of mechanical ventilation (8.4 ± 5.2 vs. 4.0 ± 4.3 days; p = 0.01) and ICU stay were observed in EnCAP patients.

**Conclusions:**

EnCAP is a severe infection which is more consistent with HCAP than with typical CAP. This retrospectively suggests that the application of HCAP guidelines should have improved EnCAP management.

## Background

*Haemophilus influenzae*, *Klebsiella pneumoniae *and *Escherichia coli *are the most common aetiological agents of community-acquired pneumonia (CAP) caused by Gram-negative bacteria (GNB) [1,2]. Epidemiological monitoring of CAP was started in 2002 in our intensive care unit (ICU) and revealed an increase in occurrence of severe *Enterobacter *CAP (EnCAP) from 2002 to 2005. No specific reference to *Enterobacter *spp. is made in CAP studies [3,4], except for their low incidence in one recent study [5]. The primary aim of this study was to describe the characteristics of EnCAP, particularly to determine their specific characteristics in comparison with CAP due to common bacteria. This comparison included the presence of criteria for health-care-associated pneumonia (HCAP) described since 2005, i.e. after completion of EnCAP cases. HCAP refer to a new category of pneumonia apparently developing in the community with the particularity to apply to patients who have recently interfaced with the health care system [6,7]. Bacteria responsible for HCAP can share the same susceptibility profile than hospital-acquired bacteria. The awareness of these criteria would then potentially improve the adequacy of empirical treatment and the prognosis of the pneumonia. In our study, we tested the hypothesis that it would have improved the specific prognosis of EnCAP.

## Methods

### Study design

The study was performed in a 16-bed medical ICU (800 admissions/year) of a French teaching hospital (Bordeaux University Hospital, Bordeaux, France). Prospective epidemiological monitoring, including all patients admitted for CAP, was initiated on 01/01/2002. From that time until 31/12/2004 (over 3 years), cases of microbiologically-confirmed EnCAP were gathered and described. Each eligible case of EnCAP was then matched retrospectively by an investigator blinded to the outcome or other characteristics with three documented controls selected from the prospective 3-year CAP cohort (excluding *Enterobacter *and fungal pneumonia). Patients with a neutrophil count of <500/mm^3 ^and aspiration pneumonia (defined by the development of a radiographically evident infiltrate in patients with witnessed aspiration or at increased risk for oropharyngeal aspiration) were also excluded. The only matching criterion between cases and controls was the same 5-year age group. According to the Comité de Protection des Personnes Sud-Ouest et Outre Mer III (DC2010/44) and because no change was done to our ICU's usual practices, informed consent was not required but patients and/or their proxies were informed of the study's purpose. The research was conducted according to the Helsinki Declaration.

### Data collection

During the study period, charts of patients admitted for pneumonia were screened for comorbidities, predefined clinical, radiological, microbiological, laboratory data and therapeutic options. Hospital admission over the previous 3 years and, when available, time between the occurrence of the first signs and admission was also registered. Admission chest X-rays were pooled and then evaluated retrospectively by two intensive care physicians blinded to the presence of cases or controls. Simplified Acute Physiology Score (SAPS II) [8], Sequential Organ Failure Assessment (SOFA) [9] and sepsis classification [10] were characterized 24 h after admission. Empirical and definitive antibiotic choice and time between antibiotic choice and admission were monitored for each patient.

### Definitions

CAP was defined by the presence of symptoms of lower respiratory tract infection along with two of the following signs: fever (>38.3°C) or hypothermia (≤36°C), leukocytosis (>10 × 10^9 ^cells/L) or leukopenia (5 × 10^8^≤cells/L≤ 4 × 10^9^), new infiltrates on chest X-ray, in patients not hospitalized. EnCAP was confirmed if *Enterobacter *was isolated from sputum, bronchoalveolar lavage (defined by an association of >25 leukocytes/mL, <25 epithelial cells on direct examination and a threshold for bacterial positive cultures of 10^4 ^colony-forming units (CFU)/mL), pleural fluid specimens, or blood cultures. Sputum cultures were considered significant if the microorganism was isolated in pure culture and if the Gram-stained section showed >25 polymorphonuclear leukocytes and <25 tracheo-bronchial epithelial cells with a threshold for positive cultures of 10^7 ^CFU/mL. HCAP criteria comprised one of the following criteria [11,12]: (i) admission from a nursing home or other long-term nursing care facility; (ii) receiving outpatient haemodialysis, peritoneal dialysis or infusion therapy requiring regular visits to a hospital-based clinic; or (iii) prior hospitalization within the preceding 12 months [11,12]. Antimicrobial therapy given for pneumonia in the 2 days before hospital admission was termed "prior antibiotic therapy". Antimicrobial therapy in the first 24 h was considered appropriate if it contained one or more antimicrobial agent with *in vitro *activity against the causative microorganism. Acute respiratory distress syndrome (ARDS) was defined according to published criteria [13].

### Microbiological study

Investigation of pathogens in blood, fluid samples or sputum was performed by standard microbiological procedures. Identification to the species level was performed with the API 20E (Biomérieux, Marcy l'Etoile, France) or Phoenix system (BD Diagnostic, Le Pont-de-Claix, France). Antibiotic susceptibility of the isolates was determined by the disk diffusion method in MH agar medium, according to French guidelines http://www.sfm.asso.fr, or by the Phoenix system. The presence of an extended-spectrum β*-*lactamase (ESBL)-producing strain was detected by the double disk synergy test [14].

### Statistics

All data were analysed using SAS version 9.2 software (SAS Institute, Cary, NC, USA). EnCAP patients were compared to patients with CAP due to common bacteria for each characteristic (except for age which was the matching criteria) by calculating odds ratio (OR) with 95% CI using unconditional regression analysis. Explanatory variables with a p value < 0.25 were included in the multivariable model using a stepwise descending method (backward). In each model, the multicollinearity was investigated between each variable: the variable that led to the best predictive model was retained.

## Results

During the 3-year study period, 134 patients were admitted to our medical ICU from outside the hospital setting for microbiologically-documented severe pneumonia. Ten patients (7.5%) had EnCAP.

### EnCAP clinical description and radiographic findings

The general characteristics of the 10 patients as well as their severity assessment, comorbidities and clinical description are summarized in Table 1. All EnCAP patients had at least one comorbidity. Four had cancer (one chronic myeloid leukaemia, one colon cancer, one pulmonary cancer and one breast cancer). All cases of EnCAP were severe as shown by SAPS II score, the occurrence of septic shock and the incidence of acute renal failure. Alveolar (20%), interstitial (30%) or mixed alveolo-interstitial (30%) aspects were observed in EnCAP. Bilateral infiltrates were common (60%). Pleural involvement was observed in 10% of cases of EnCAP.

**Table 1:** General characteristics and description of patients with community-acquired pneumonia due to *Enterobacter sp*. compared to community-acquired-pneumonia due to common bacteria

	*Enterobacter *CAP (n = 10)	CAP (n = 30)
Age (years)	59.8 ± 20	64.3 ± 15.3
Male/female, %	80/20	64/36
SAPS II	44.9 ± 11.2	39 ± 13.3
SOFA/organ failure*	5.7 ± 3.3/1.2 ± 1.1	4.9 ± 3.3/0.9 ± 1.1
Comorbidities, n	1.7 ± 0.7	0.9 ± 0.8
COPD	4	12
cancer or haematological disease	4	6
diabetes mellitus	4	5
neurological	3	3
chronic heart failure	1	3
chronic renal failure	1	2
At least one comorbidity, %	100	60
Smoking history, n	3	11
Time between onset of symptoms and admission, days	3 ± 2	3 ± 2.7
Symptoms, n		
T°>37.5°C	7	22
highest temperature, °C	39.6 ± 0.6	39.0 ± 1.0
chills	1	5
sweating	1	0
cough + sputum	4	16
Acute onset**^$ ^**/Progressive onset, n	4/6	19/11
Sepsis classification, n (%)		
sepsis	2 (20)	10 (33)
severe sepsis	3 (30)	12 (40)
septic shock	5 (50)	8 (27)
Leukocytosis (G/L)	10.2 ± 4.8	15.1 ± 7.0
C-reactive protein (mg/dL)	207 ± 150	202 ± 132
Blood urea (mmol/L)	17.9 ± 12	10.3 ± 9.9
ARDS, n (%)	8 (80)	14 (47)
At least one HCAP criterion ^¤^, n	10	5

*Organ failure was defined by a SOFA score of 3 or higher for the evaluated parameter (example: a respiratory failure was considered if respiratory SOFA was 3 or 4); **^$^**acute onset: <24 h; ^¤^cf. method

CAP community-acquired pneumonia; SAPS Simplified acute physiology score; SOFA: sequential organ failure assessment; COPD: chronic obstructive pulmonary disease; ARDS: acute respiratory distress syndrome; HCAP: Health-care associated pneumonia

### EnCAP bacteriological findings

*E. cloacae *and *E. aerogenes *were confirmed in seven and three cases, respectively. The microbiological diagnosis was established by culture of sputum (4/10), bronchoalveolar lavage (3/10) or tracheobronchial aspirates in ventilated patients (3/10) and by blood cultures in one case. In one patient, *E. cloacae *was isolated (1.5 × 10^6 ^CFU/mL) in association with *Moraxhella catarrhalis *(10^4 ^CFU/mL) from bronchoalveolar lavage. ESBL-producing strains were isolated in four cases, including ciprofloxacin resistance in two, gentamicine resistance in one and amikacine resistance in another strain.

### Univariate analysis of factors associated with CAP due to *Enterobacter sp *compared with CAP due to common bacteria

Each case of EnCAP was compared with three controls so that 30 patients with common CAP were included. A description of both groups and the results of univariate analysis are shown in table 1 and 2 respectively. In the control group, 34 bacterial species were recovered: *Streptococcus pneumoniae *(n = 8), methicillin-susceptible *Staphylococcus aureus *(n = 6), *H. influenzae *(n = 5), *Legionella pneumophilia *and *E. coli *(n = 4), *Streptococcus viridans *(n = 3), *Chlamydia pneumoniae *(n = 2), *Klebsiella oxytoca *and *K. pneumoniae *(n = 1

**Table 2:** Factors associated with community-acquired pneumonia due to *Enterobacter sp*. in comparison with community-acquired pneumonia due to common bacteria

	Univariate analysis		Multivariate analysis	
	Odds ratio^£^ (95% confidence interval)	p	Odds ratio^£^ (95% confidence interval)	p
Male Sex	0.33 (0.06-1.81)	0.20	...	
SAPS II	1.04 (0.98-1.1)	0.21	...	
SOFA	1.08 (0.87-1.34)	0.5	...	
Organ failure*	1.25 (0.65-2.4)	0.5	...	
Comorbidities			...	
0-1	1	0.02		
2-3	4.13 (0.92-18.52)			
Time between onset of symptoms and admission	1 (0.74-1.34)	0.97	...	
Highest temperature	2.07 (0.84-5.14)	0.12	...	
Progressive onset Acute onset**^$^**	1 0.39 (0.09-1.67)	0.2	...	
Sepsis classification				
Sepsis	1	0.4		
Severe sepsis	1.25 (0.17-9.02)			
Septic shock	3.13 (0.47-20.58)			
Leukocytosis (G/L)	0.87 (0.76-1)	0.05	0.75 (0.59-0.96)	0.02
C-reactive protein (mg/dL)	1 (0.95-1.01)	0.92	...	
Blood urea (mmol/L)	1.06 (1-1.13)	0.07	...	
Radiographic findings			...	
Predominant alveolar	1	0.41		
Predominant interstitiel	1.85 (0.43-7.96)			
ARDS	0.22 (0.04-1.21)	0.08	...	
Criteria for HCAP				
No criterion	1	<0.01	1	<0.01
At least one criterion	45 (4.61-439.16)		244.6 (7.48-999.99)	

**^$ ^**Odds ratios were adjusted for age * Organ failure was defined by a SOFA score of 3 or higher for the evaluated parameter; **^$^**acute onset: <24 h

HCAP: Health-care associated pneumonia; SAPS: simplified acute physiology score; SOFA: sequential organ failure assessment; ARDS: acute respiratory distress syndrome

#### Clinical and biological findings

No difference was observed regarding age. By univariate analysis, EnCAP patients tended to present with more comorbidities (particularly diabetes and cancer or haematological disease) while common CAP patients had a more important leukocytosis. EnCAP patients also tended to be more severe than CAP patients, as shown by the incidence of acute renal failure and ARDS.

#### Radiographic findings

There was no significant difference in radiographic findings

#### Criteria for HCAP

All patients in the EnCAP group were classified retrospectively as HCAP as prior hospitalization within the preceding 12 months was observed in all cases. The mean time between previous and current hospital admission was 2.7 ± 3.9 months (range: 0.5-12 months). All patients were living at home but one was considered as a home care patient since he received intravenous therapy at home. One patient had two HCAP criteria (chronic renal failure requiring haemodialysis).

#### Multivariate analysis

Only a lower leukocytosis and the presence of at least one criterion for HCAP were associated with EnCAP vs. CAP due to common bacteria.

#### Antimicrobial therapy and sepsis outcome

Empirical antimicrobial therapy did not differ significantly between the two groups (Table 3): a single antibiotic was used in 4/10 cases of EnCAP and 7/30 cases of CAP (β-lactam drug in all cases but one, a macrolide for the ultimate patient). A β-lactam drug combined with a macrolide or fluoroquinolone was used in all other patients in both groups. For EnCAP, empirical therapy was appropriate in only 20% of patients compared to 97% for CAP (p < 0.01). Clinical improvement was delayed, as demonstrated by 2 additional days between the initiation of empirical antimicrobial therapy at hospital admission and subsequent definitive appropriate antimicrobial therapy (p < 0.01). A trend in extended overall length of antimicrobial therapy was observed for EnCAP compared to CAP patients. After final antimicrobial therapy reassessment, 8/10 patients received a combination of antibiotics (including four with aminoglycosides) for EnCAP compared to 19/30 CAP patients. The length of mechanical ventilation and ICU stay were increased for EnCAP compared to CAP, with only a trend towards a delayed resolution of septic shock (as measured by the length of vasoactive drug use) and an increase in hospital mortality.

**Table 3:** Antimicrobial therapy, other treatments and outcome of patients with community-acquired pneumonia due to *Enterobacter sp*. compared to community-acquired-pneumonia due to common bacteria

	*Enterobacter *CAP (n = 10)	CAP (n = 30)	p value
Prior antimicrobial treatment*, %	70	37	0.08
Prior antimicrobial treatment appropriateness**^σ^**, %	14	46	0.19
Empirical antimicrobial treatment appropriateness^$^, %	20	97	< 0.01
Time between hospital admission**^£ ^**and definite appropriate antimicrobial treatment (days)	3.3 ± 1.6	1.2 ± 0.6	<0.01
Time between onset of empirical antimicrobial therapy and apyrexia (days)**^¤^**	5.6 ± 2.1	3.8 ± 2.4	0.06
Length of antimicrobial treatment (days)	11.8 ± 5.2	9.6 ± 3.8	0.16
Vasoactive or inotropic drug length of use (days)**	8 ± 3.9	4.8 ± 3.2	0.13
Dialysis, n (%)	3 (30)	3 (10)	0.14
Ventilation^¤¤^, n (%)	10 (100)	18 (60)	0.96
Length of ventilation (days)	8.4 ± 5.2	4 ± 4.3	0.01
Length of ICU stay (days)	21 ± 15	11.9 ± 9.2	0.04
Hospital mortality, n (%)	3 (30)	5 (17)	0.37

Data are expressed as mean ± SD unless specified otherwise.

*Antibiotic started <24 h before ICU admission; **^σ^**prior antimicrobial treatment was defined as a treatment initiated in the 2 days preceding hospital admission; **^$^**empirical antimicrobial treatment was initiated after hospital admission as soon as pneumonia was diagnosed; **^£^**at admission for CAP; ^**¤**^apyrexia was defined as temperature ≤37°C; **for five and eight patients respectively in EnCAP and CAP; ^¤¤^invasive or non-invasive ventilation.

SD: standard deviation; CAP: community-acquired pneumonia; ICU: intensive care unit.

## Discussion

This study provides new insights into non-nosocomial *Enterobacter *pneumonia where previously published data are scarce. This type of pneumonia is severe and seems to develop in patients with serious comorbidities. Bouza et al. [15] performed a retrospective study of 50 cases of *Enterobacter *bacteraemia developing in the community and reported several results that are in agreement with our own conclusions: (i) a high prevalence of underlying comorbidities; (ii) severe clinical presentation; and (iii) high incidence of septic shock (30% in their study, 50% in ours) [15]. Broughton et al. [16] reported an original case of *E. cloacae *necrotizing CAP. In our study, a predominant alveolo-interstitial pattern was observed with no cavitation or abscesses. This was reported previously nearly 30 years ago in a reference radiographic study [17]. No single clinical or radiographic finding other than a lower leukocytosis was specific enough to consider this pathogen in pneumonia.

However, these results should not conceal another important study suggestion, that misclassifying pneumonia occurring outside the hospital setting as CAP rather than HCAP could have a number of consequences on outcome. A recent editorial highlighted the fact that HCAP criteria remained poorly identified, the majority of physicians persisting in the prescription of regimens consistent with CAP recommendations [18]. This should be particularly crucial in patients with severe pneumonia admitted to the ICU. In 2007, an extension of previously proposed criteria was proposed [11] and validated more recently [12]. Until and probably since 2005, patients presenting with pneumonia outside the hospital setting admitted to hospital or the ICU escaped these recommendations [18-20]. We sought to retrospectively seek for HCAP criteria because in our ICU we faced with an increase in incidence of pneumonia due to an unusual community-acquired bacterial species (i.e. *Enterobacter *sp.) before 2005. Moreover we observed that *Enterobacter *spp. were more frequent in epidemiological HCAP studies [11,18,19] than in CAP studies [3-5]. Indeed, patients received empirical antibiotic treatments designed to take into account typical CAP bacteria, but these antibiotics failed to treat most EnCAP episodes (80%). A delay in the improvement of EnCAP was observed in comparison with adequately treated CAP. Should HCAP criteria have been acknowledged, empirical antibiotics with broader spectrum would have been used and an improved prognosis would have probably occurred. In the EnCAP group, the most relevant criterion that led to the retrospective diagnosis of HCAP was a history of previous hospitalization. The recent proposition of extension to >1 year [11] confers the advantage of improving the negative predictive value of HCAP criteria in order to make physicians more confident about using antibiotics with activity limited to purely community flora. Extending the interval beyond 1 year would reduce the positive predictive value without further improving the negative predictive value, and broad-spectrum antibiotic treatment would then be used unnecessarily. Among the criteria for HCAP, a history of previous hospitalization or antibiotic treatment is less easy to retrieve than admission from a health-care facility, especially in the ICU where patients often cannot be questioned. Our study shows that history of previous hospitalization must be investigated thoroughly in patients presenting with non-nosocomial pneumonia, or HCAP will potentially be wrongly classified as CAP.

The high proportion (40%) of ESBL-producing *Enterobacter *spp. in our study was significant. The emergence of ESBL enterobacteriaceae (ESBLE) strains in the community has been described previously, particularly in Europe [21]. Known risk factors include diabetes, prior hospital admission and previous antibiotic use [22] which corroborate our results. Of note, aminoglycosides remain often effective against these strains. Whether ESBLE gastrointestinal carriage is due to selection by antibiotic treatment used in the community, or to prolonged nosocomial acquisition in the community, remains unclear. Oral third-generation cephalosporins are commonly used in outpatients in France. Third-generation cephalosporins are known risk factors for ESBLE carriage [23]. However, in a more recent study, nosocomial acquisition of ESBLE followed by prolonged digestive carriage prior to community-acquired infection was strongly suggested by genotypic analysis [24].

The small population size in our study is an important limitation. Any conclusions drawn from this study should be regarded with caution. Besides, one could argue that BAL also recovered *Moraxhella catarrhalis *in a patient from the EnCAP group. However, BAL is a highly specific method and *Enterobacter sp*. grew to a higher threshold than *Moraxhella sp*. (1.5 × 10^6 ^CFU/mL vs 10^4 ^CFU/mL). Moreover the empiric antibiotic treatment of this patient (i.e. coamoxiclav) covered *Moraxhella sp*. but not *Enterobacter sp*. explaining the delay in the improvement of clinical signs of CAP. Statistical results excluding this case were not modified. The study precludes any firm conclusions about the relationship between the delay in appropriate antibiotic therapy and its influence on outcome. The severity of the underlying illnesses could have played an independent role and only multivariate analysis would have provided a sounder insight into this complex relationship. However, it is now accepted that any delay in appropriate antibiotic treatment in patients with septic shock is associated with a worse outcome [25]. The design of our study does not answer the question as to whether the worse outcome of EnCAP compared to common CAP is due to antibiotic inappropriateness (i.e. noncompliance with HCAP concept) or to *Enterobacter *itself. Only a comparison between *Enterobacter *HCAP and EnCAP could answer this question. However, two studies have reported the absence of specific findings with *Enterobacter *sp. compared with other Gram-negative bacteria [1,17]. Therefore, it suggests that only noncompliance with HCAP concept was responsible for this worse outcome. Finally, previous antibiotic treatment could not be assessed accurately in our patients as it was a retrospective study. Some of the patients in the EnCAP group could also have received antibiotic treatment in the previous months. However, as already suggested, this is not always easy to ascertain in the real-life management of severe pneumonia.

## Conclusions

In conclusion, this study shows that intensive care and other physicians can be faced with severe EnCAP in patients with comorbidities. The study is a plea for a greater awareness of the HCAP criteria: to ignore them may lead to inappropriate antibiotic treatment. HCAP criteria, and particularly history of previous hospital admission, should be systematically and thoroughly investigated at the time of admission of such patients.

## Competing interests

The authors declare that they have no competing interests.

## Authors' contributions

AB conceived the study, participated in its design and in acquisition of data, coordinated the study and wrote the article. BA participated in the statistical analysis and in the article redaction. FV participated in the acquisition of patients' data and in the conception of the study. MY carried out the acquisition of data. SMT participated in the statistical analysis. VD and CB coordinated the bacteriological study and participated in the article redaction. AMR participated in the design of the study and in the article redaction. DG conceived the study, participated in the design of the study and in the article redaction. As a whole, all authors read and approved the final manuscript.

## Pre-publication history

The pre-publication history for this paper can be accessed here:

http://www.biomedcentral.com/1471-2334/11/120/prepub
